# Four New Species of *Hemileccinum* (Xerocomoideae, Boletaceae) from Southwestern China

**DOI:** 10.3390/jof7100823

**Published:** 2021-09-30

**Authors:** Mei-Xiang Li, Gang Wu, Zhu L. Yang

**Affiliations:** 1 CAS Key Laboratory for Plant Diversity and Biogeography of East Asia, Kunming Institute of Botany, Chinese Academy of Sciences, Kunming 650201, China; limeixiang@mail.kib.ac.cn (M.-X.L.); wugang@mail.kib.ac.cn (G.W.); 2 Yunnan Key Laboratory for Fungal Diversity and Green Development, Kunming Institute of Botany, Chinese Academy of Sciences, Kunming 650201, China; 3 College of Life Sciences, University of Chinese Academy of Sciences, Beijing 100049, China

**Keywords:** boletes, taxonomy, morphology, molecular phylogeny

## Abstract

The genus *Hemileccinum* belongs to the subfamily Xerocomoideae of the family Boletaceae. In this study, phylogenetic inferences of *Hemileccinum* based on sequences of a single-locus (ITS) and a multi-locus (nrLSU, *tef1-α*, *rpb1*, *rpb2*) were conducted. Four new species, namely *H. abidum*, *H. brevisporum*, *H. ferrugineipes* and *H. parvum* were delimited and proposed based on morphological and molecular evidence. Descriptions and line-drawings of them were presented, as well as their comparisons to allied taxa. Our study shed new light on the recognition of the genus. The pileipellis of the species in this genus should mostly be regarded as (sub)epithelium to hyphoepithelium, because the pileipellis of most studied species here is composed of short inflated cells in the inner layer (subpellis) and filamentous hyphae in outer layer (suprapellis). The basidiospores of the studied species, including the type species, *H. impolitum*, have a warty surface.

## 1. Introduction

The genus *Hemileccinum* Šutara was created based on the species *H. impolitum* (Fr.) Šutara as the type, and *H. depilatum* (Redeuilh) Šutara [1]. These two species were both originally placed in the genus *Boletus* L. [2] and later were transferred to the genus *Leccinum* because of the lateral stipe stratum of the leccinoid type which is predominantly anticlinally arranged, breaking up into characteristic fascicles of hyphae ending in elements of the caulohymenium during growth of the stipe [1,3,4]. However, molecular phylogenetic analyses indicated that these two species are very distant from the species of both *Leccinum* and *Boletus*, but similar to the species of *Xerocomus*; thus they were accordingly transferred to *Xerocomus* [5,6]. Based on the previous molecular evidence and his own further morphological observations, Šutara established the genus *Hemileccinum* to accommodate these two species. He emphasized that *Hemileccinum* was diagnosed by the anatomical structure of the peripheral stipe layers having a lateral stipe stratum of the leccinoid type, which distinguished this genus not only from all the other species belonging to the *Xerocomus* s.l. but also from those in the genus *Boletus* [1]. Wu et al. confirmed the monophyly of *Hemileccinum* and found an additional diagnosed character of this genus, namely the irregularly warty basidiospores under SEM [7,8]. Meanwhile, the genus *Corneroboletus* N.K. Zeng & Zhu L. Yang was confirmed as a synonym of *Hemileccinum* due to the similar basidiospore ornamentation and the closely phylogenetic relationship [8].

As ectomycorrhizal fungi, species in the genus *Hemileccinum* are widely distributed in temperate, subtropical and tropical regions, and play an important role in forest ecology [1,8,9,10,11,12,13,14]. However, the species diversity of *Hemileccinum* was relatively poorly known in the world until recent studies suggested existence of other potentially new specie of the genus [8,10]. Until now, only 10 species of the genus have been reported in the world according to the database of INDEX FUNGORUM (accessed date: 27 August 2021). Among them, *H. impolitum* and *H. depilatum* are from Europe [1], *H.* *subglabripes* (Peck) Halling, *H. rubropunctum* (Peck) Halling and *H. hortonii* (A.H. Sm. & Thiers) M. Kuo & B. Ortiz are from North America [8,9]. In Asia, *H. rugosum* G. Wu & Zhu L. Yang is described from China, and *H. indecorum* (Massee) G. Wu & Zhu L. Yang is from tropical China, Singapore, Thailand [8,15,16].

During past fungal investigations in southwestern China, we encountered four potential new species of *Hemileccinum*. Our aim in this study is to clarify their molecular phylogenetic positions and to delimit them based on morphological data and molecular evidence.

## 2. Materials and Methods

### 2.1. Sample Collection and Morphological Study

**Figure 1:**
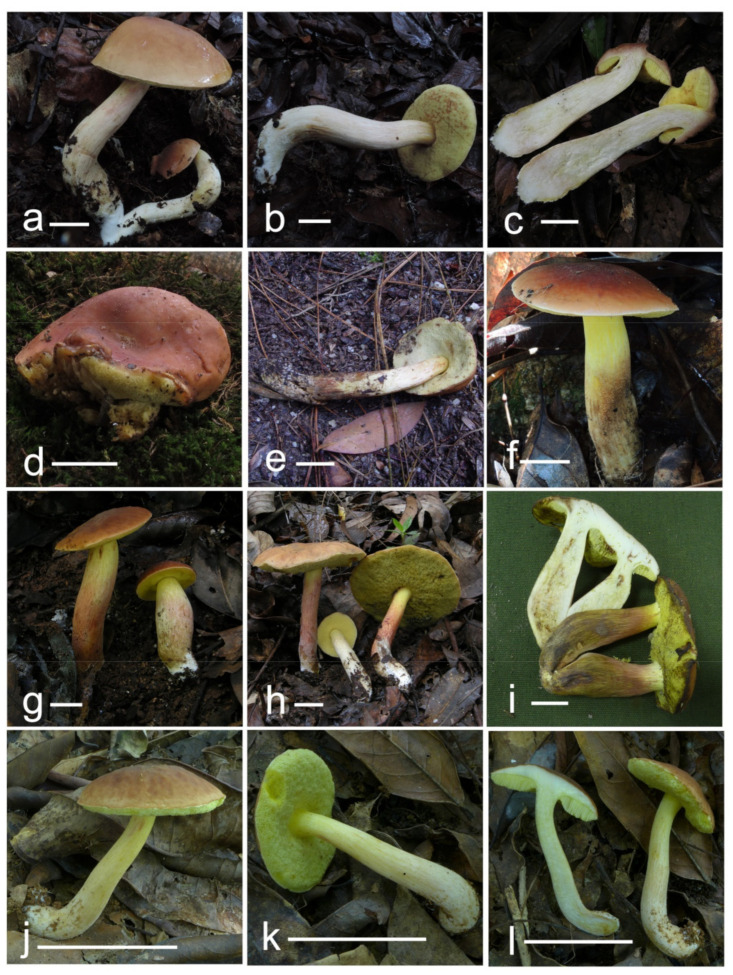
Fresh basidiomata of *Hemileccinum* species. (**a**–**c**) *H. albidum* ((**a**,**b**) Type, KUN-HKAS81120, (**c**) KUN-HKAS87225); (**d**–**f**) *H. brevisporum* ((**d**) KUN-HKAS67896, (**e**) KUN-HKAS59445, (**f**) Type, KUN-HKAS89150); (**g**–**i**) *H. ferrugineipes* ((**g**,**h**) Type, KUN-HKAS115554, (**i**) KUN-HKAS75054); (**j**–**l**) *H. parvum* ((**j**–**l**) Type, KUN-HKAS115553) Bars = 30 mm.

In total, seventeen collections were examined in this study, which were collected from the Yunnan Province of southwestern China during the years 2007–2017 (Figure 1). The macroscopic characters of the specimens were described based on fresh basidiomata, and the dried specimens were deposited in the Cryptogamic Herbarium of the Kunming Institute of Botany, Chinese Academy of Sciences (KUN-HKAS). Color codes of the form “5C4” indicate the plate, row, and color block from Kornerup and Wanscher [17]. For microscopic observation, a ZEISS Axiostar Plus microscope (Oberkochen, Germany) was used and the dried specimens were revived in 5% KOH solution or distilled water. Moreover, Melzer’s reagent was applied to test color reactions of the tissue fragments to the solution. Microscopic studies follow Li et al. and Zhou et al. [18,19]. In the descriptions of basidiospores, the abbreviation [n/m/p] means n basidiospores measured from m basidiomata of p collections. The range notation (a)b–c(d) stands for the dimensions of basidiospores in which b–c contains a minimum of 90% of the measured values while a and d in the brackets stand for the extreme values. Q is used to imply “length/width ratio” of a basidiospore in side view; Q_m_ means average Q of all basidiospores ± sample standard deviation. To observe basidiospore ornamentations, a ZEISS Sigma 300 scanning electron microscope (SEM) (Oberkochen, Germany) was used. Genera are abbreviated as follows: *H.* for *Hemileccinum*, Ca. for *Castanopsis*, *C.* for *Castanea*, *L.* for *Lithocarpus*, *P.* for *Pinus*, *Q.* for *Quercus* and *Rug.* for *Rugiboletus*.

### 2.2. Molecular Procedures and Phylogenetic Analyses

Total genomic DNA was obtained with the Ezup Column Fungi Genomic DNA Purification Kit (Sangon Biotech, Shanghai, China) according to the manual from material dried with silica gel. A total of five nuclear loci were sequenced, including the internal transcribed spacer (ITS), the large subunit of nuclear ribosomal RNA gene (nrLSU), the polymerase II subunit one (*rpb1*) gene, the second largest subunit of RNA polymerase II (*rpb2*), and the translation elongation factor 1-α gene (*tef1-α*). The primer pairs of ITS1/ITS4 [20,21], LROR/LR5 [22,23] were used for amplifying ITS, nrLSU, respectively. The primer pairs used for amplifying the *rpb1*, *rpb2*, *tef1-α*, followed those in Wu et al. [7]. PCR was performed in a total volume of 25 μL containing 1 μL forward primer, 1 μL reverse primer, 9.5 μL nuclease-free H_2_O, 12.5 μL BlasTaq^TM^ 2×PCR MasterMix (abm, Richmond, VA, Canada) and 1 μL DNA template. PCR protocol was as follows: pre-denaturation at 95 °C for 5 min, followed by 35 cycles of denaturation at 95 °C for 60 s, 52 °C for 60 s, and 72 °C for 80 s, and then a final elongation at 72 °C for 8 min was included. The PCR products were purified with a Gel Extraction and PCR Purification Combo Kit (Spin-column) (Bioteke, Beijing, China), and then sequenced by ABI-3730-XL DNA Analyzer (Applied Biosystems, Foster City, CA, USA) by using the same primer pairs as in the PCR amplification for sequencing.

### 2.3. Phylogenetic Analyses

We used BLAST to compare the obtained sequences of the newly collected materials with those in the GenBank database. The BLAST results were used to predict the phylogenetic relationship between the newly collected specimens and known species and indicated that the new materials were genetically similar to the other species of *Hemileccinum*. In this study, two datasets were produced, the ITS dataset, and the combined nrLSU, *tef1-α*, *rpb1* and *rpb2* dataset. The ITS sequences of *Hemileccinum* species from China were used to infer relationships of Chinese species with those from Europe, North America and East Asia. In the analysis of ITS dataset, *Phylloporus rubrosquamosus* N.K. Zeng, Zhu L. Yang & L.P. Tang, *Phylloporus rubeolus* N.K. Zeng, Zhu L. Yang & L.P. Tang, *Hourangia cheoi* (W.F. Chiu) Xue T. Zhu & Zhu L. Yang and *Hourangia pumila* (M.A. Neves & Halling) Xue T. Zhu, Halling & Zhu L. Yang were chosen as outgroup [24,25,26]. The combined dataset was mainly used to infer phylogenetic relationships and systematic positions of the Chinese species. In the multigene phylogenetic analysis, including all known genera in the subfamily Xerocomoideae were included. We screened the relevant sequences deposited in GenBank, which were mainly submitted by Wu et al. [7,8], Gelardi et al. [27], Zhu et al. [26], Zeng et al. [24], Neves et al. [28]. We collected a total of 13 ingroup species of 8 known genera within Xerocomoideae and 2 outgroup species outside Xerocomoideae but in the Boletaceae. Detailed information of the voucher specimens is given in Table 1.

The sequences were assembled with SeqMan implemented in Lasergene v7.1 (DNASTAR Inc., Madison, WI, USA), and then aligned by using MAFFT v7.310 [29]. The software Bioedit v7.2.5 [30] was used to check aligned matrices. To assess any potential conflicts in the gene tree topologies for these five loci, single-locus matrices were analyzed using maximum likelihood (ML) in RAxML v8.0.20 [31]. Sequences of the loci without conflicts were then concatenated using Phyutility 2.2 [32,33] for further phylogenetic analyses. The best-fitted substitution model for each gene was determined through MrModeltest v2.4 [34] by using Akaike Information Criterion (AIC). GTR + I + G was inferred as the best-fit model for the nrLSU, *tef1-α*, *rpb1* and ITS selected according to the AIC in MrModeltest v2.4 [34]. SYM + I + G was selected as the best model for *rpb2*. For the ultimate phylogenetic analyses, Maximum Likelihood (ML) analysis and Bayesian Inference were conducted by RAxML v8.0.20 [31] and MRBAYES v3.2.7 [35], respectively. The parameters of RAxML were set as defaults with 500 bootstrap replicates, except the substitution model which was set as GTRGAMMAI.

BI analyses were conducted with two independent runs of one cold and three heated chains. Runs were performed for 2 million generations, and trees sampled every 100 generations. The convergence was determined with the average standard deviation of split frequencies (<0.01) Chain convergence was determined using Tracer v1.5 to confirm sufficiently large ESS values (>200). The sampled trees were subsequently summarized by using the “sump” and “sumt” commands with a 25% burn-in [31,35]. The Bayesian posterior probabilities (BPP) of internodes were estimated based on the majority rule consensus with the remaining trees.

**Table 1:** Specimens used in phylogenetic analysis and their GenBank accession numbers. The newly generated sequences are shown in bold.

Species	Voucher	Locality	GenBank Accession Number					References
			ITS	nrLSU	*rpb2*	*rpb1*	*tef1-α*	
*Hemileccinum rugosum*	KUN-HKAS84355	China	-	KT990578	KT990413	KT990931	KT990774	[8]
*Hemileccinum rugosum*	KUN-HKAS84970	China	-	KT990577	KT990412	-	KT990773	[8]
*Hemileccinum rugosum*	KUN-HKAS50284	China	-	KT990576	KT990411	-	KT990772	[8]
*Hemileccinum subglabripes*	MICH:KUO-07230802	USA	-	MK601738	MK766300	-	MK721092	[10]
*Hemileccinum subglabripes*	MICH:KUO-07070702	USA	-	MK601737	MK766299	-	MK721091	[10]
*Hemileccinum subglabripes*	MICH:KUO-08301402	USA	-	MK601739	MK766301	-	MK721093	[10]
*Hemileccinum subglabripes*	72206	USA	-	KF030303	-	KF030374	KF030404	[36]
*Hemileccinum subglabripes*	294169	USA	MN128237	-	-	-	-	from GenBank
*Hemileccinum subglabripes*	3660	-	KM248936	-	-	-	-	from GenBank
*Hemileccinum depilatum*	2137333	USA	AY127032	-	-	-	-	from GenBank
*Hemileccinum depilatum*	AF2845	Belgium	-	-	MG212633	-	MG212591	[37]
*Hemileccinum depilatum*	Bd1	-	-	AF139712	-	-	-	[5]
*Hemileccinum impolitum*	Bim 1	Germany	-	AF139715	-	KF030375	JQ327034	[36]
*Hemileccinum impolitum*	47698	Portugal	AJ419187	-	-	-	-	[38]
*Hemileccinum impolitum*	BI57407	Thailand	KM235997	-	-	-	-	from GenBank
*Hemileccinum impolitum*	BI57408	Thailand	KM235998	-	-	-	-	from GenBank
*Hemileccinum impolitum*	17173	USA	JF907783	-	-	-	-	[39]
*Hemileccinum impolitum*	KUN-HKAS84869	Germany	-	KT990575	KT990410	KT990930	KT990771	[8]
*Hemileccinum indecorum*	KUN-HKAS63126	China	-	KF112440	-	-	-	[7]
*Hemileccinum indecorum*	OR0863	Thailand	-	-	MH614772	-	MH614726	[16]
*Hemileccinum rubropunctum*	JLF56666	USA	MH190826	-	-	-	-	from GenBank
*Hemileccinum rubropunctum*	MES256	USA	FJ480428	-	-	-	-	[40]
*Hemileccinum rubropunctum*	NY-792788REH-8501	USA	-	MK601768	MK766327	-	MK721122	[10]
*Hemileccinum rubropunctum*	NY-01193924REH-9597	USA	-	MK601769	MK766328	-	MK721123	[10]
*Hemileccinum* sp.	KUN-HKAS53421	China	-	KF112432	KF112751	KF112565	KF112235	[7]
*Hemileccinum hortonii*	MICH KUO-07050706	USA	-	MK601821	MK766377	-	MK721175	[10]
***Hemileccinum albidum***	KUN-HKAS87225	China	MZ923777	MZ923774	MZ936317	MZ936334	MZ936351	**This study**
***Hemileccinum albidum***	KUN-HKAS83355	China	MZ923778	MZ923775	MZ936321	MZ936340	MZ936357	**This study**
***Hemileccinum albidum* (T) **	KUN-HKAS81120	China	MZ923782	MZ923766	MZ936320	MZ936339	MZ936352	**This study**
***Hemileccinum albidum***	KUN-HKAS50503	China	MZ923781	MZ923767	MZ936319	MZ936335	MZ936355	**This study**
***Hemileccinum albidum***	KUN-HKAS50350	China	MZ923779	MZ923768	MZ936323	MZ936342	MZ936359	**This study**
***Hemileccinum albidum***	KUN-HKAS84554	China	MZ923780	-	MZ936318	MZ936336	MZ936358	**This study**
***Hemileccinum albidum***	KUN-HKAS85753	China	MZ923786	-	MZ936325	MZ936337	MZ936353	**This study**
***Hemileccinum albidum***	KUN-HKAS87105	China	-	MZ923769	MZ936327	MZ936338	MZ936356	**This study**
***Hemileccinum albidum***	KUN-HKAS83333	China	MZ923784	-	MZ936326	MZ936344	MZ936361	**This study**
***Hemileccinum albidum***	KUN-HKAS83400	China	MZ923783	MZ923770	MZ936324	MZ936341	MZ936354	**This study**
***Hemileccinum albidum***	KUN-HKAS115749	China	MZ923785	-	MZ936322	MZ936343	MZ936360	**This study**
***Hemileccinum brevisporum* (T) **	KUN-HKAS89150	China	MZ923788	MZ923764	MZ936328	MZ936345	MZ936362	**This study**
***Hemileccinum brevisporum***	KUN-HKAS59445	China	-	KT990579	KT990414	KT990932	KT990775	[8]
***Hemileccinum brevisporum***	KUN-HKAS67896	China	MZ923787	MZ923765	MZ936329	MZ936346	MZ936363	**This study**
***Hemileccinum ferrugineipes* (T) **	KUN-HKAS115554	China	MZ923792	MZ923773	MZ936330	MZ936350	MZ973011	**This study**
***Hemileccinum ferrugineipes***	KUN-HKAS75054	China	-	KF112377	KF112749	KF112563	KF112234	[7]
***Hemileccinum ferrugineipes***	KUN-HKAS93310	China	MZ923791	-	MZ936331	MZ936347	MZ973012	**This study**
***Hemileccinum parvum***	KUN-HKAS99764	China	MZ923789	MZ923771	MZ936332	MZ936349	MZ973009	**This study**
***Hemileccinum parvum* (T) **	KUN-HKAS115553	China	MZ923790	MZ923772	MZ936333	MZ936348	MZ973010	**This study**
*Heimioporus* sp.	KUN-HKAS53451	China	-	KF112345	KF112805	KF112616	KF112226	[7]
*Heimioporus* aff. *japonicus*	KUN-HKAS52236	China	-	KF112346	KF112807	KF112617	KF112227	[7]
*Heimioporus japonicas*	KUN-HKAS52237	China	-	KF112347	KF112806	KF112618	KF112228	[7]
*Aureoboletus tenuis*	KUN-HKAS75104	China	-	KT990518	KT990359	KT990897	KT990722	[8]
*Aureoboletus thibetanus*	KUN-HKAS76655	China	-	KF112420	KF112752	KF112626	KF112236	[7]
*Pulchroboletus roseoalbidus*	AMB 12757	Italy	-	NG_060126	-	-	KJ729512	[27]
*Alessioporus ichnusanus*	AMB 12756	Italy	-	NG_057044	-	-	KJ729513	[27]
*Phylloporus rubrosquamosus*	KUN-HKAS52552	China	-	KF112391	KF112780	-	KF112289	[7]
*Phylloporus rubrosquamosus*	KUN-HKAS54559	China	NR120124	NG_042668	-	-	JQ967175	[24,25]
*Phylloporus rubeolus*	KUN-HKAS52573	China	JQ967259	JQ967216	-	-	JQ967172	[24,25]
*Xerocomus fraternus*	KUN-HKAS55328	China	-	KT990681	KT990497	-	KT990869	[8]
*Xerocomus velutinus*	KUN-HKAS68135	China	-	KT990673	-	KT991011	KT990861	[8]
*Hourangia cheoi*	Yang 5153	China	KP136997	KP136947	KP136975	KP136966	KP136924	[26]
*Hourangia pumila*	REH8063	Indonesia	JQ003626	NG_060636	-	-	-	[28]
*Boletellus indistinctus*	KUN-HKAS77623	China	-	KT990531	KT990371	-	KT990733	[8]
*Boletellus indistinctus*	KUN-HKAS80681	China	-	KT990532	KT990368	KT990903	KT990734	[8]
*Leccinum variicolor*	KUN-HKAS57758	China	-	KF112445	KF112725	KF112591	KF112251	[7]
*Leccinum* aff. *scabrum*	KUN-HKAS57266	China	-	KF112442	KF112722	KF112590	KF112248	[7]
*Leccinum monticola*	KUN-HKAS76669	China	-	KF112443	KF112723	KF112592	KF112249	[7]
*Leccinellum cremeum*	KUN-HKAS90639	China	-	-	KT990420	KT990936	KT990781	[8]
*Leccinellum* sp.	KUN-HKAS53410	China	-	KT990585	KT990421	KT990937	-	[8]

## 3. Results

### 3.1. Molecular Phylogenetic Analysis

**Figure 2:**
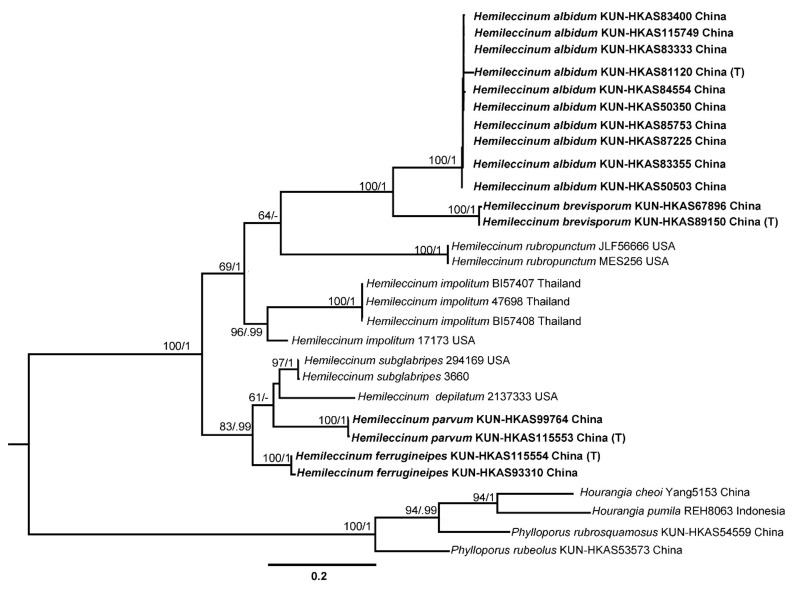
Maximum-Likelihood phylogenetic tree generated from ITS dataset. Bootstrap values (BP) ≥ 50% from ML analysis and Bayesian posterior probabilities (PP) ≥ 0.90 are shown on the branches. Newly described species are indicated in bold and their type specimens are marked with (T).

A total of 79 sequences, including 16 for ITS, 12 for nrLSU, 17 for *tef1-α*, 17 for *rpb1*, and 17 for *rpb2* were newly generated in the present study and aligned with sequences downloaded from GenBank and previous studies. Sequences retrieved from GenBank and obtained in this study were listed in Table 1. ML and BI analyses of the ITS dataset resulted in almost identical topologies and thus only the tree inferred from ML analysis was displayed (Figure 2). Our phylogenetic analyses indicated that *Hemileccinum* formed a monophyletic group with evident support (MLB/BPP = 100%/1.0). Eight phylogenetic species of the genus *Hemileccinum* were retrieved, and four of them could be new to science.

**Figure 3:**
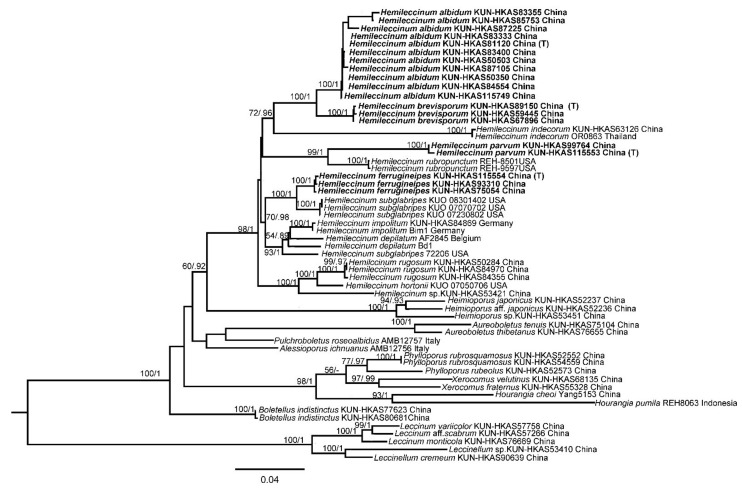
Maximum-Likelihood analysis of *Hemileccinum* with nrLSU, *tef1-α*, *rpb1* and *rpb2* sequence data. Bootstrap values (BP) ≥ 50% from ML analysis and Bayesian posterior probabilities (PP) ≥ 0.90 are shown on the branches. Newly described species are indicated in bold and their type specimens are marked with (T).

According to the four single-locus phylogenetic analyses, no strongly supported (>70% of ML) conflict of topologies was observed. Therefore, sequences of the four DNA loci were concatenated for the final analysis. ML and BI analyses of the concatenated data set resulted in almost identical topologies and thus only the tree inferred from ML analysis was displayed (Figure 3). Our molecular phylogenetic analysis indicated that *Hemileccinum* is a monophyletic genus with high statistic supports (BP = 98%, PP = 1). Thirteen phylogenetic species of the genus *Hemileccinum* were retrieved, and four of them could be new to science. By further morphological examinations of the related specimens of those four potential new species, we verified their taxonomic statuses of new species. For detailed information of each species, see below.

### 3.2. Taxonomy

**Figure 4:**
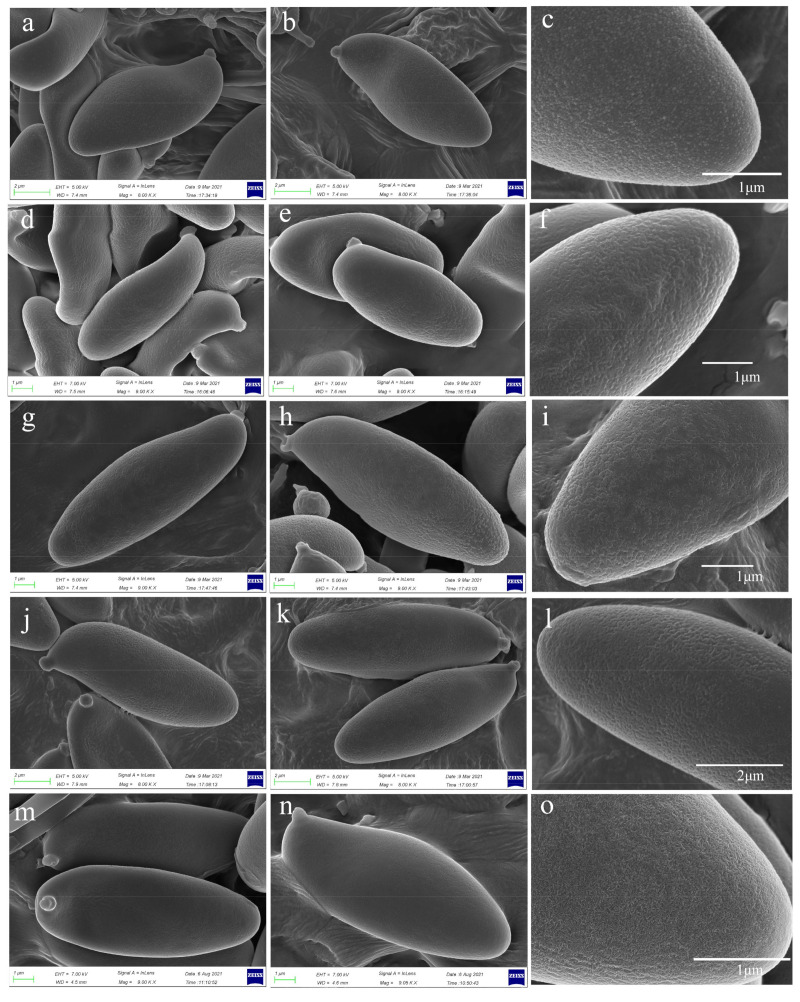
Basidiospores of *Hemileccinum albidum*, *H. brevisporum*, *H. ferrugineipes*, *H. parvum* and *H. impolitum* under SEM. (**a**–**c**) *H. albidum* (Type, KUN-HKAS81120); (**d**–**f**) *H. brevisporum* (Type, KUN-HKAS89150); (**g**–**i**) *H. ferrugineipes* (Type, KUN-HKAS115554,); (**j**–**l**) *H. parvum* (Type, KUN-HKAS115553); (**m**–**o**) *H. impolitum* (KUN-HKAS84869).

**Figure 5:**
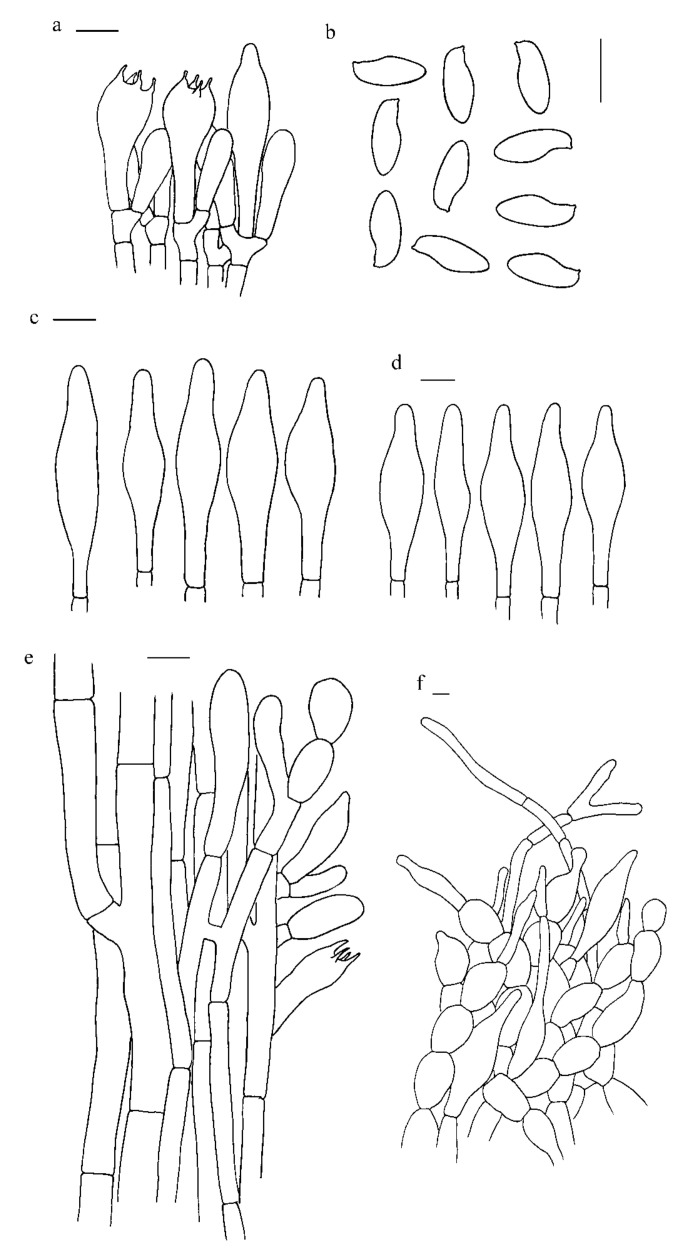
Microscopic features of *H. albidum* (Type, KUN-HKAS81120). (**a**). Hymenium and subhymenium; (**b**). Basidiospores; (**c**). Cheilocystidia; (**d**). Pleurocystidia; (**e**). Stipitipellis; (**f**). Pileipellis. *Bars*: a = 20 µm, b = 30 µm, c–e = 20 µm, f = 10 µm.

***Hemileccinum albidum*** Mei-Xiang Li, Zhu L. Yang & G. Wu, **sp**. **nov**., Figure 1a–c, Figure 4a–c and Figure 5.

MycoBank no: 840704

*Etymology*: The epithet ‘*albidum*’ refers to the somewhat white stipe of this species.

*Type*: CHINA. Yunnan Province: Jingdong County, Ailao Mt., alt. 2490 m, associated with *Castanopsis ceratacantha*, Ca. *rufescens*, *Lithocarpus xylocarpus*, *Quercus griffithii*, 22 July 2013, Jiao Qin 682 (KUN-HKAS81120).

*Diagnosis*: *Hemileccinum albidum* is distinguished by the combination characters of the even pileus, and the whitish, nearly smooth stipe, with only small, granular scales at the base.

*Description*: *Basidioma* stipitate-pileate, small to medium-sized. *Pileus* 3–9 cm diam, hemispherical to applanate, finely rugose, then smooth, finely subtomentose, dry or slightly viscid when wet; surface of *Pileus* grey-brown when young, then chrome yellow (5A8), pompeian yellow (5C7) to ochraceous (2D2–5) or golden brown (5D7), somewhat paler along the pileus edge; context white (1A1), yellowish (2A4–5) to brownish (5B5–8, 6C6–8), unchanging on exposure. Hymenophore depressed around the apex of the stipe; hymenophoral surface yellowish (2A4–5) to yellow to sulphur yellow (4A5–4A6) or olivaceous yellow (2A6–7), unchanging when bruised; pores roundish, 0.5–1.0(1.5)/mm; tubes up to 10 mm long, concolorous with the hymenophoral surface, unchanging when bruised. *Stipe* 5–16 cm long, 1.0–2.5 cm wide, subcylindrical; surface whitish (8A1), cream (1A2) to pale yellow-brown (2A3) or pinkish (13A2) to purplish (11B3–5), fibrillose, sometimes covered with small pale granular scales; context unchanging in color when cut. *Basal mycelium* white (1A1).

*Basidia* 25–38 × 10–14 µm, clavate, 4-spored, sterigmata 4–6 µm long. *Basidiospores* [120/3/3] (10)11–12.5 × (4.0)4.5–5.5 µm, [Q = (2.00)2.18–2.66(2.75), Q_m_ = 2.36 ± 0.12], subfusiform in side view with distinct suprahilar depression, subfusoid in ventral view, brownish yellow, inamyloid, with tiny warts and pinholes on the surface under SEM. *Hymenophoral trama* nearly phylloporoid with hyphae of the lateral strata touching or almost touching each other with hyphae diverging from the central strand to the subhymenium. *Cheilocystidia* 41–50 × 8–11 µm, lanceolate to clavate or ventricose, thin-walled, colorless. *Pleurocysitidia* 46–56 × 8–13 µm, ventricose-subfusiform, with long beak, thin-walled. *Pileipellis* an hyphoepithelium 170–230 µm thick, composed of moniliform hyphal segments 5–37 µm wide, thin-walled, with narrowly cylindrical to shortly cystidioid terminal cells 10–75 × 3–20 µm. *Pileal trama* composed of interwoven hyphae 5–34 µm wide. *Stipitipellis* ca. 130 µm thick, hymeniform, terminal cells broadly clavate, 20–43 × 10–22 µm, sometimes connected with narrow, filamentous hyphae at the outer layer. *Caulocystidia* abundant, 26–43 × 7–12 µm, thin-walled. *Stipe trama* composed of parallel hyphae, 3.5–12.0 µm wide. *Clamp connections* absent in all tissues.

*Habitat and distribution*: Scattered in subtropical forests dominated by plants of the family Fagaceae (*Castanopsis ceratacantha*, Ca. *rufescens*, Ca. *calathiformis*, *Lithocarpus xylocarpus*, *L. hancei*, *L. mairei* and *Quercus griffithii*); on acidic, loamy, humid soils; moderately common in southwestern China; fruiting in June to August in southwestern China (Yunnan Province) between 1968 and 2490 m altitude.

*Additional specimens examined*: CHINA. Yunnan Province: Jingdong County, Ailao Mt., alt. 2490 m, associated with *Castanopsis ceratacantha*, Ca. *rufescens*, *Lithocarpus xylocarpus* and *Quercus griffithii*, 21 July 2006, Zhu-Liang Yang 4706 (KUN-HKAS50503); same location, 20 July 2006, Yan-Chun Li 596 (KUN-HKAS50350); same location, 23 July 2013, Bang Feng 1359 (KUN-HKAS115749); Longling County, Zhenan Town, alt. 1968 m, associated with *Castanopsis calathiformis* and *Lithocarpus hancei*, 11 July 2014, Xiao-Bin Liu 459 (KUN-HKAS87105); same location, 22 July 2014, Xiao-Bin Liu 673 (KUN-HKAS87225); same location, 25 August 2014, Chen Yan 155 (KUN-HKAS85753); Longling County, Xueshan Village, alt. 2000 m, associated with *Castanopsis ceratacantha*, *Lithocarpus mairei* and *Quercus griffithii*, 19 June 2014, Jiao Qin 916 (KUN-HKAS83333); same location, 21 June 2014, Jiao Qin 938 (KUN-HKAS83355); same location, 31 July 2014, Jiao Qin 983 (KUN-HKAS83400); same location, 14 June 2014, Li-Hong Han 258 (KUN-HKAS84554).

*Notes*: *Hemileccinum albidum* is distinguished by combination characters of the even pileus and whitish stipe surface covered with concolorous, small granular scales. Phylogenetically, *H. albidum* is closely related to *H.* *brevisporum*. However, the former species differs in its whitish stipe and larger basidiospores (11.0–12.5 × 4.5–5.5 μm). Morphologically, the size, pileus color and shape of *H. albidum* are similar to those of the European *H. depilatum*. However, the latter is different from the former by its wrinkled or hammered pileus and the pileipellis composed of hyphae of spherical and shortly cylindrical, terminal cells 16.5–44.0 × 8.5–30.0 μm [4]. Ecologically, *H. albidum* occurs under trees of Fagaceae in subtropical regions while *H. depilatum* is distributed in hardwoods, especially with trees of *Ulmus* and *Carpinus* in temperate regions [41,42] (Appendix A).

**Figure 6:**
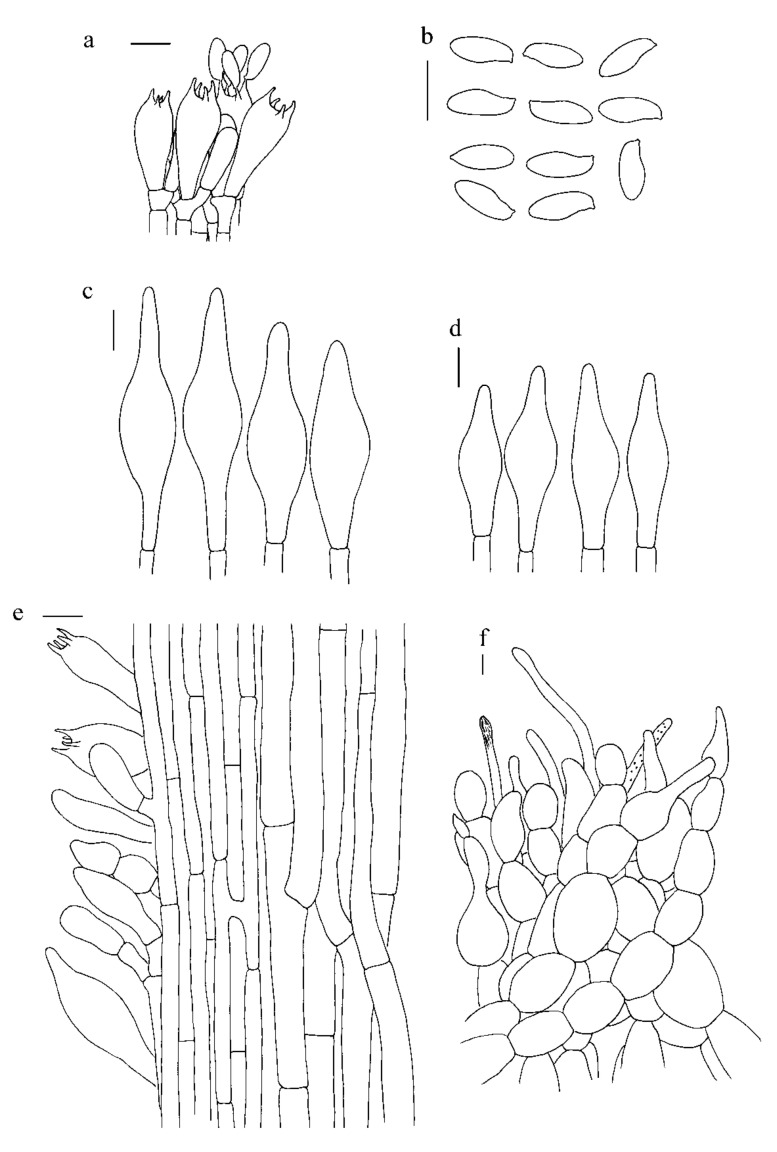
Microscopic features of *H. brevisporum* (Type, KUN-HKAS89150). (**a**). Hymenium and subhymenium; (**b**). Basidiospores; (**c**). Pleurocystidia; (**d**). Cheilocystidia; (**e**). Stipitipellis; (**f**). Pileipellis. *Bars*: a = 20 µm, b = 30 µm, c–e = 20 µm, f = 10 µm.

***Hemileccinum brevisporum*** Mei-Xiang Li, Zhu L. Yang & G. Wu, **sp**. **nov**., Figure 1d–f, Figure 4d–f and Figure 6.

MycoBank no: 840701

*Etymology*: The epithet ‘*brevisporum*’ refers to the short basidiospores.

*Type*: CHINA. Yunnan Province: Menghai County, alt. 1700 m, associated with *Castanopsis calathiformis*, Ca. *indica* and *Lithocarpus truncates*, 1 July 2014, Kuan Zhao 487 (KUN-HKAS89150).

*Diagnosis*: Differs from other *Hemileccinum* species by the combined characters of the dense fine-grained scales on the stipe surface, the shorter basidiospores measuring 9–11 × 4–5 µm and small basidia measuring 18.5–27.0 × 8–11 µm.

*Description*: *Basidioma* stipitate-pileate, fleshy, small to medium-sized. *Pileus* 9 cm diam, glabrous to slightly subtomentose, dry, convex to planate, pale yellow-brown (2A3) to pale red-brown (7A5); context yellowish (3A5–3A6), unchanging when bruised. Hymenophoral surface and tubes concolorous, flash yellow (3A8) to dull yellow (3B3–3B4), unchanging when bruised, pores roundish, 1.0–1.5/mm, tubes 11 mm long, unchanging when injured. *Stipe* 13–15 cm long, 2.0–2.3 cm wide, subcyclindrical, surface of stipe cream (2A2–3A2) to yellowish (2A4–2A5) at upper part, pale yellow-brown to yellow-brown (6C8) at lower part, covered with small yellowish brown (5D8) dotted scales, context of stipe cream to pale yellow (1A2–1A3), unchanging when bruised. *Basal mycelium* white to cream (2A2–3A2).

*Basidia* 18.5–27.0 × 8–11 µm, clavate, hyaline in 5% KOH, 4-spored. *Basidiospores* [80/2/2], 9–11 × 4–5 µm, [Q = (2.22)2.35–2.50(2.75), Q_m_ = 2.37 ± 0.15], subfusiform and inequilateral in side view with distinct suprahilar depression, subfusoid in ventral view, yellowish to brownish, smooth under light microscopy, but with tiny warts on the surface under SEM. *Hymenophoral trama* nearly phylloporoid with hyphae of the lateral strata touching or almost touching each other with hyphae diverging from the central strand to the subhymenium; hyphae subcylindrical to cylindrical, 3.5–14.0 µm wide. *Cheilocystidia* 37–50 × 11–13 µm, ventricose-subfusiform, with long beak, thin-walled. *Pleurocystidia* 48–67 × 12–16 µm, ventricose subfusiform, with long beak, thin-walled. *Pileipellis* an hyphoepithelium 150–210 µm thick, composed of moniliform hyphal segments 5–35 µm wide, thin-walled, with narrowly cylindrical to shortly cystidioid terminal cells 6–53 × 4–20 µm. *Pileal trama* composed of interwoven hyphae 5–37 µm wide. *Stipitipellis* ca. 100 µm thick, hymeniform, terminal cells broadly clavate, 13–30 × 7.0–12.5 µm, sometimes connected with narrow, filamentous hyphae at the outer layer. *Caulobasidia* abundant, 18.5–28.0 × 9.0–12.0 µm, thin-walled. *Stipe trama* composed of parallel hyphae, 4–12 µm wide. *Clamp connections* absent.

*Habitat and distribution*: Scattered in subtropical forests dominated by the families Fagaceae (*Castanopsis calathiformis*, Ca. *indica*, Ca. *orthacantha*, *Lithocarpus hancei*, *L. mairei* and *Quercus griffithii*) and Pinaceae (*Pinus yunnanensis* or *P. armandii*); on acidic or slightly alkaline, loamy soils; rather rare; fruiting in July to August in southwestern to northwestern Yunnan between 1700 and 2120 m altitude.

*Additional specimens examined*: CHINA. Yunnan Province: Longling County, alt. 2010 m, associated with *Castanopsis calathiformis*, *Lithocarpus hancei* and *Pinus yunnanensis*, 9 July 2009, Yan-Chun Li 1698 (KUN-HKAS59445); Jianchuan County, Laojunshan town, alt. 2120 m, associated with *Castanopsis orthacantha*, *Lithocarpus mairei*, *Quercus griffithii* and *Pinus armandii*, 9 August 2010, Qing Cai 334 (KUN-HKAS67896).

*Notes*: *Hemileccinum brevisporum* is morphologically similar to *H. impolitum* because of the ornamentation in the stipe and the slightly subtomentose pileus surface [1,41,42]. However, *H. impolitum*, originally described from Europe, differs from *H. brevisporum*, by its much stockier stipe, and larger basidiospores (12–15 × 4–6 μm). Ecologically, *H. brevisporum* occurs under trees of Fagaceae and Pinaceae in subtropical regions while *H. impolitum* is distributed in hardwood or floodplain forests, especially with trees of *Quercus* and *Fagus* in temperate regions [41,42] (Appendix A).

**Figure 7:**
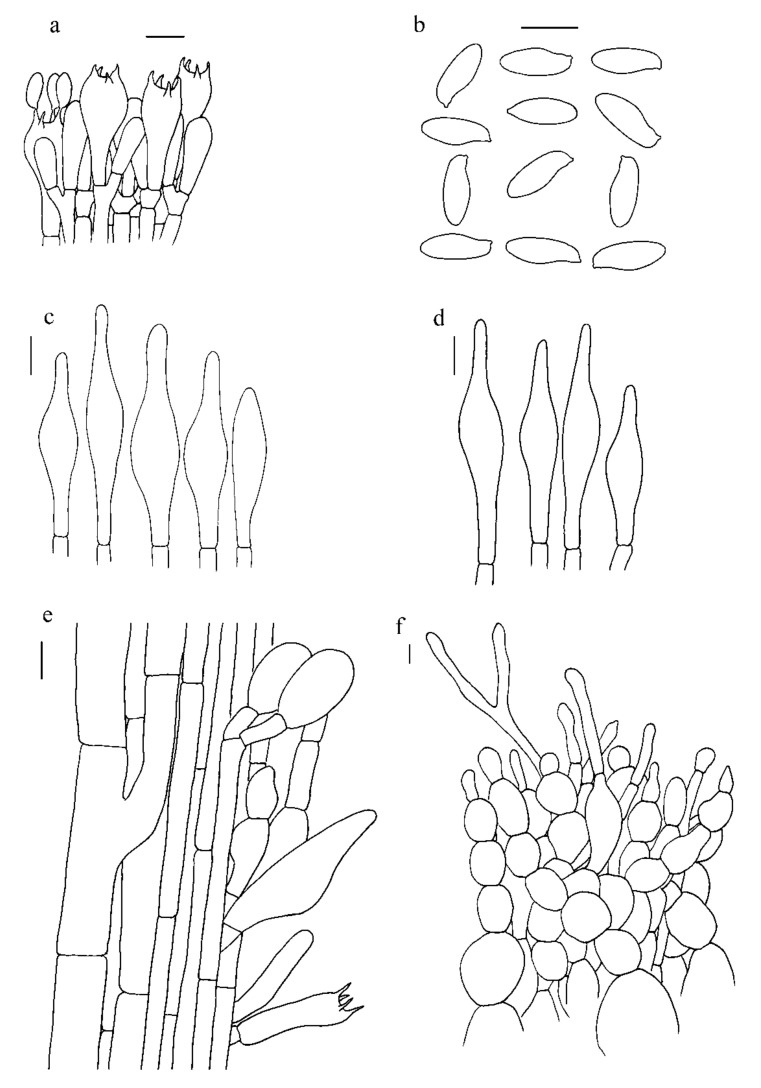
Microscopic features of *H. ferrugineipes* (Type, KUN-HKAS115554). (**a**). Hymenium and subhymenium; (**b**). Basidiospores; (**c**). Pleurocystidia; (**d**). Cheilocystidia; (**e**). Stipitipellis; (**f**). Pileipellis. *Bars*: a = 20 µm, b = 30 µm, c–e = 20 µm, f = 10 µm.

***Hemileccinum ferrugineipes*** Mei-Xiang Li, Zhu L. Yang & G. Wu, **sp**. **nov**., Figure 1g–i, Figure 4g–i and Figure 7.

MycoBank no: 840700

*Etymology*: The epithet ‘*ferrugineipes*’ refers to the reddish brown stipe of this species.

*Type*: CHINA. Yunnan Province: Pu’er City, Simao District, Taiyanghe Nature Reserve, alt. 1200 m, associated with *Castanopsis ferox*, Ca. *calathiformis*, *Cyclobalanopsis xanthotricha*, *Quercus fabri*, *Q. variabilis* and *Lithocarpus glabra*, 24 June 2016, Jian-Wei Liu 584 (KUN-HKAS115554).

*Diagnosis*: Differs from other *Hemileccinum* species by the combined characters of rugose pileus, creamy yellow stipe surface when young becoming reddish when mature, and densely scaled surface of the stipe.

*Description*: *Basidioma* stipitate-pileate, small to medium-sized. *Pileus* 3–10 cm diam, clavate to planate, surface rugose, slightly subtomentose, dry, yellowish brown (5E5), olive brown (4E5–6) to dull brown (5E8–5F8), context cream to yellowish (2A4–5), unchanging when bruised. Hymenophoral surface and tubes concolorous, yellow (1A2–1A3) to ochreous (5B7–5C7), unchanging when bruised, pores roundish, 1.5–2.5/mm; tubes 4–6 mm long, unchanging when injured. *Stipe* 4–10 cm long, 1–2 cm wide, subcylindrical, surface yellowish to yellow at upper part, lower part pale red-brown of stipe pileus; covered with longitudinal striations and densely dotted scales, context cream (1A2) to yellowish, unchanging when bruised. *Basal mycelium* cream.

*Basidia* 23–35 × 9–13 µm, clavate, hyaline in 5% KOH, 4-spored. *Basidiospores* [80/2/2], 11.0–12.5 × 4–5 µm, [Q = (2.30)2.40–2.78(3.00), Q_m_ = 2.63 ± 0.19], subfusiform in side view with distinct suprahilar depression, ellipsoid to somewhat oblong in face view, yellowish to brownish, smooth under light microscopy, but with tiny warts under SEM. *Hymenophoral trama* nearly phylloporoid with hyphae of the lateral strata touching or almost touching each other with hyphae diverging from the central strand to the subhymenium; hyphae subcylindrical to cylindrical, 4–13 µm wide. *Cheilocystidia* 36–63 × 7–11 µm, ventricose-subfusiform, with long beak, thin-walled. *Pleurocystidia* 37–62 × 8–12 µm, ventricose subfusiform, with long beak, thin-walled. *Pileipellis* an hyphoepithelium 170–270 µm thick, composed of moniliform hyphal segments 5–42 µm wide, thin-walled; always with narrowly cylindrical to shortly cystidioid terminal cells, 20–127 × 4–12 µm. *Pileal trama* composed of interwoven hyphae 6–38 µm wide. *Stipitipellis* ca.30–40 µm thick, hymeniform, terminal cells broadly clavate, 15.0–32.0 × 6.5–15.5 µm. *Caulobasidia* abundant, 28–44 × 9–12 µm, thin-walled. *Stipe trama* composed of parallel hyphae, 5–13 µm wide. *Clamp connections* absent.

*Habitat and distribution*: Scattered in subtropical forests dominated by plants of the family Fagaceae (*Castanopsis ferox*, Ca. *calathiformis*, Ca. *hystrix*, *Cyclobalanopsis xanthotricha*, *Quercus fabri*, *Q. variabilis* and *Lithocarpus glabra*); on acidic or slightly alkaline, loamy soils; rather rare; fruiting in June to August in southwestern to northwestern Yunnan between 1200 and 1690 m altitude.

*Additional specimens examined*: CHINA. Yunnan Province: Baoshan City, Longyang District, alt. 1690 m, associated with *Castanopsis calathiformis*, *Quercus fabri* and *Lithocarpus glabra*, 30 July 2017, Pan-Meng Wang 350 (KUN-HKAS93310); Lanping County, alt. 1400 m, associated with *Castanopsis hystrix*, *Quercus fabri* and *Lithocarpus glabra*, 16 August 2011, Gang Wu 759 (KUN-HKAS75054).

*Notes*: *Hemileccinum ferrugineipes* is characterized by its rugose pileus and small, dense, dotted scales on the reddish-brown stipe. Phylogenetically, the American species *H. subglabripes* is close to *H. ferrugineipes*, but differs from it by its fairly long and slender, nearly smooth stipe [9,10,43,44,45]. Morphologically, *H. ferrugineipes* is similar to *Rugiboletus extremiorientalis* (Lj.N.Vassiljeva) G. Wu & Zhu L. Yang and *H. hortonii* in the rugose pileus and dense scales on the stipe [44,45,46,47]. However, *H. ferrugineipes* differs from *Rug. extremiorientalis*, originally described from subtropical Yunnan, China, by its reddish slightly densely scaled surface of the stipe and hyphoepithelium pileipellis. *H. ferrugineipes* differs from *H. hortonii*, originally described from Illinois, USA, by its tightly wrinkled pileus and the stockier stipe [9,44,45]. Ecologically, *H. ferrugineipes* occurs under trees of Fagaceae in subtropical regions; *H. hortonii* is scattered or in groups on the ground under mixed deciduous woods, occasionally under conifers; *H. hortonii* is rather rare and might be found in eastern North America, west to Michigan [44,45] (Appendix A).

**Figure 8:**
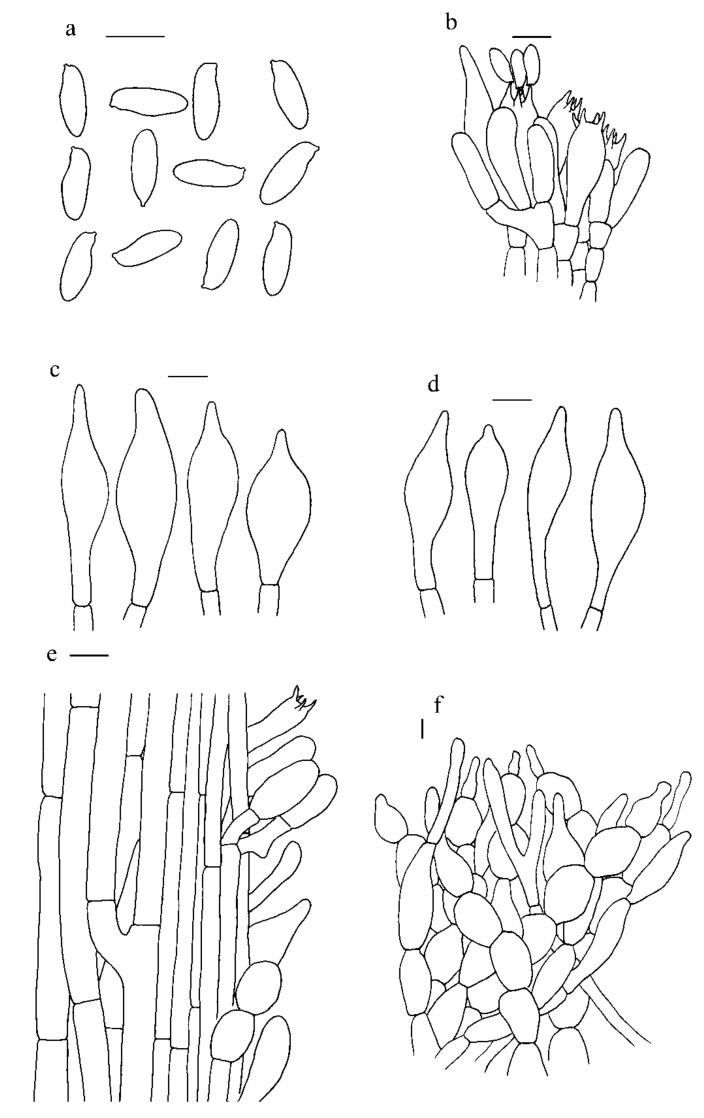
Microscopic features of *H. parvum* (Type, KUN-HKAS115553) (**a**). Basidiospores; (**b**). Hymenium and subhymenium; (**c**). Pleurocystidia; (**d**). Cheilocystidia; (**e**). Stipitipellis; (**f**). Pileipellis. *Bars*: a = 30 µm, b–e = 20 µm, f = 10 µm.

***Hemileccinum parvum*** Mei-Xiang Li, Zhu L. Yang & G. Wu, **sp**. **nov**., Figure 1j–l, Figure 4j–l and Figure 8.

MycoBank no: 840703

*Etymology*: The epithet ‘*parvum*’ refers to the small basidioma.

*Type*: CHINA. Yunnan Province: Wenshan City, Malipo County, alt. 1200 m, associated with *Castanea henryi*, *C. mollissima*, *Lithocarpus bonnetii* and *Quercus marlipoensis*, 30 July 2017, 532624MF-201-Wu 2299 (KUN-HKAS115553).

*Diagnosis*: Differs from other *Hemileccinum* species by the combined characters of the small basidioma, and the rugose surface of pileus, the pale yellow context staining pale blue very slowly when bruised.

*Description*: *Basidioma* stipitate-pileate, small. *Pileus* 3.3–3.6 cm diam, rugose, slightly subtomentose, hemispherical, brownish (5B5–8, 6C6–8) at the central part, becoming paler towards the margin (brownish or yellowish); context pale yellow (1A2–1A3), staining pale blue very slowly when bruised at some spots, 4–5 mm thick. Hymenophoral surface and tubes concolorous, light yellow (3B4–3B5), pores roundish, 1.5–2.0/mm, unchanging when bruised; tubes 4–5 mm long, sinuate near the stipe. *Stipe* 6.0–9.7 cm long, 0.4–0.9 cm wide, clavate, central, solid, pale yellow (2A2–2A4) at the upper part and becoming paler downwards, surface ornamented with coarsely small squamules; context light yellow (3B4–3B5), unchanging when bruised. *Basal mycelium* white (1A1).

*Basidia* 20.5–32.0 × 8.0–10.5 µm, clavate, 4-spored; sterigmata up to 4–5 µm long. *Basidiospores* [80/2/2], 12–14 × 4.5–5.0 µm, [Q = (2.40)2.50–2.80(2.88), Q_m_ = 2.69 ± 0.11], subfusiform and inequilateral in side view with distinct suprahilar depression, subfusoid in ventral view, yellowish to brownish, inamyloid, smooth under light microscopy, but with tiny warts on the surface under SEM. *Hymenophoral trama* phylloporoid with hyphae of the lateral strata touching or almost touching each other with hyphae diverging from the central strand to the subhymenium; hyphae subcylindrical to cylindrical, 4–12 µm wide. *Cheilocystidia* 41–50 × 8–11 µm, lanceolate to clavate or ventricose, thin-walled, colorless. *Pleurocysitidia* 45–65 × 9–11 µm, ventricose-subfusiform, with long beak, thin-walled. *Pileipellis* an hyphoepithelium 160–240 µm thick, composed of moniliform hyphal segments 6–30 µm wide, thin-walled, with narrowly cylindrical to shortly cystidioid terminal cells 10–87 × 5–17 µm. *Pileal trama* composed of interwoven hyphae 6–30 µm wide. *Stipitipellis* ca. 100 µm thick, hymeniform, terminal cells broadly clavate, 20.0–43.0 × 10.0–21.5 µm, sometimes connected with narrow, filamentous hyphae at the outer layer. *Caulocystidia* abundant, 24.5–60.0 × 10.5–19.0 µm, thin-walled. *Stipe trama* composed of parallel hyphae, 3.5–12.0 µm wide. *Clamp connections* absent in all tissues.

*Habitat and distribution*: Scattered in subtropical forests dominated by plants of the family Fagaceae (*Castanea henryi*, *C. mollissima*, *Lithocarpus bonnetii* and *Quercus marlipoensis*); on acidic, wet, fertile soils; rather rare; fruiting in July in southeastern Yunnan between 1200 and 1300 m altitude.

*Additional specimens examined*: Yunnan Province: Wenshan City, Malipo County, alt. 1300 m, associated with *Castanea henryi*, *C. mollissima*, *Lithocarpus bonnetii* and *Quercus marlipoensis*, 27 July 2016, Gang Wu 1645 (KUN-HKAS99764).

*Notes*: *Hemileccinum parvum* is morphologically similar to *H. subglabripes* because of the slightly wrinkled pileus and the slender stipe [9,48], However, *H. subglabripes*, originally described from the USA, differs from *H. parvum* by the nearly smooth stipe of the latter covered with branny particles on the stem which are pale and easily overlooked, and the larger basidioma. Our data show that *H. parvum* is phylogenetically close to *H. rubropunctum*, but the latter differs by its longer stipe and the red scales on it [10,44,45]. Ecologically, *H. parvum* occurs under trees of Fagaceae in subtropical southeastern Yunnan; *H. subglabripes* inhabits mixed deciduous trees, sometimes under spruce in eastern and particularly northern North America; and *H. rubropunctum* grows in mixed woods with oak or chestnut in northeastern North America [44,45] (Appendix A).

## 4. Discussion

The genus *Hemileccinum* Šutara is geographically widely distributed, but its species diversity is poorly known. In Asia, only two species have been previously reported with molecular evidence. One is *H. indecorum* from tropical areas, and the other is *H. rugosum* from subtropical Yunnan, China [8,15]. In this study, four new species in China were recognized and delimited. They are well-supported by molecular phylogenetic and morphological evidence. The host specificity, altitude and edaphic factors seem to be important for determining the distribution of different species of *Hemileccinum*. Our newly described species are distributed in the broad-leaved and mixed forests in southwestern China. *Hemileccinum albidum* and *H. brevisporum* are distributed on high altitudes: between 1700 and 2500 m a.s.l., while *H. ferrugineipes*: 1200–1700 m a.s.l., and *H. parvum*: 1200-1300 m a.s.l. *Hemileccinum albidum*, *H. ferrugineipes* and *H. parvum* are found in subtropical forests and associated with plants of the family Fagaceae (*Castanopsis ceratacantha*, Ca. *rufescens*, Ca. *ferox*, Ca. *hystrix*, Ca. *calathiformis*; *Castanea henryi*, *C. mollissima*; *Cyclobalanopsis xanthotricha*; *Lithocarpus xylocarpus*, *L. hancei*, *L. mairei*, *L. glabra*, *L. bonnetii*; *Quercus griffithii*, *Q. fabri*, *Q. variabilis*, *Q. marlipoensis*), growing mostly on acidic soils. However, *H. ferrugineipes* can also be found in slightly alkaline habitats. *Hemileccinum brevisporum* is found in subtropical broad-leaved and mixed forests, growing with members of Fagaceae (*Castanopsis calathiformis*, Ca. *indica*, Ca. *orthacantha*; *Lithocarpus hancei*, *L. mairei*; *Quercus griffithii*) and Pinaceae (*Pinus yunnanensis* or *P. armandii*) on acidic or slightly alkaline soils. The species we described here are hardly seen in the wild mushroom market, thus their edibility is unknown yet. However, referring to the edibility of the European/American species of *Hemileccinum* [41,42,43,44,45,49], the newly described species could also be edible, but we need more investigations to confirm this.

Overall, the proposed new species are significantly different from the Asian species *H. indecorum* because the viscid pileus and stipe of the latter species are densely covered with whitish to dirty white, small conical to subconical to irregular squamules [15]. They also quite differ from the European species *H. impolitum*, which has a relatively bald pileal surface and a collapsed trichoderm pileipellis when mature [1]. Šutara reported that the basidiospores of *H. impolitum* are smooth [1]. Our re-examination of European material of *H. impolitum* under SEM indicated that there are irregular warts on the surface of basidiospores as with those of other species in *Hemileccinum* (Figure 4m–o). Accordingly, *H. depilatum* (Redeuilh) Šutara should also have a warty basidiospore surface.

The description of the new species also sheds new light on the recognition of the genus. The pileipellis of the species in this genus should mostly be regarded as (sub)epithelium to hyphoepithelium, because the pileipellis of most studied species here are composed of short inflated cells in the inner layer (subpellis) and filamentous hyphae in outer layer (suprapellis), with *H. indecorum* standing at one extremity with whitish to dirty white, small conical to subconical to irregularly shaped squamules on the pileus surface [15] and *H. impolitum* located at the other extremity with collapsed trichoderm pileipellis when mature [1]. The lateral stipe stratum of *H. impolitum* in this genus was diagnosed as the leccinoid type, predominantly anticlinally arranged hyphae ending in elements of the caulohymenium [1,3,4]. However, on the basis of the observation on our new species, this feature is not present in all species of *Hemileccinum*. The structure of the lateral stipe stratum is traceable in our species.

Based on the current study, we increased the species diversity of the genus *Hemileccinum* from Asia and reconstructed a comprehensive phylogenetic tree which included almost all known species of this genus. However, probably due to the limitations of species sampling or insufficient genetic variation of the DNA loci we used, the deep phylogenetic relationships within the genus remain unresolved. In future work, more species with detailed morphological observations and phylogenomic analysis will provide new evidence for these relationships.

## Data Availability

Publicly available datasets were analyzed in this study. This data can be found here: https://www.ncbi.nlm.nih.gov/; http://www.mycobank.org/; http://purl.org/phylo/treebase/phylows/study/TB2:S28729, accessed on 18 September 2021.

## Appendix A

**Key to known species of *Hemileccinum* in the world**	
1. Pileus surface pale brown, brown to reddish brown, more or less even	2
1′. Pileus surface orange to reddish orange, distinctively wrinkled	9
2. Pileus surface slightly subtomentose, without small conical to subconical to irregularly shaped squamules, known in subtropical and temperate regions in the Northern Hemisphere…	3
2′. Pileus surface with whitish to dirty white, small conical to subconical to irregularly shaped squamules, known from tropical southeast Asia…	*H. indecorum*
3. Basidioma varied in size, yellowish context without color change when bruised…	4
3′. Basidioma usually small in size (≤ 4 cm in daim), the pale yellow context staining pale blue very slowly when bruised, known from subtropical areas…	*H. parvum*
4. Stipe surface covered with obvious ornaments…	5
4′. Stipe surface covered with small scales, not obvious	6
5. Stipe surface ornamented with distinctly reddish brown longitudinal streaks, known from East Asia	*H. ferrugineipes*
5′. Stipe surface often covered with red dense fine-grained scales, known from North America…	*H. rubropunctum*
6. Basidiospores longer in length (> 11 µm), and in other parts of the world…	7
6′. Basidiospores shorter in length (≤ 11 µm), and distributed in subtropical forests in southwestern China.…	*H. brevisporum*
7. Stipe stout (> 2.5 cm in diam.); pileipellis an trichoderm, collapsed when mature, restricted to Europe	*H. impolitum*
7′. Stipe slender (≤ 2.5 cm in diam.); pileipellis an hyphoepithelium	8
8. Stipe surface whitish, covered with small, granular scales only at the base, distributed in subtropical China	*H. albidum*
8′. Stipe surface yellowish, nearly smooth, covered with indistinctive tiny scales, known from eastern North America	*H. subglabripes*
9. Basidiospores smaller (10–12 × 4–5 µm), distributed in subtropical and tropical China	*H. rugosum*
9′. Basidiospores larger (≥ 12 µm in length), distributed in temperate regions…	10
10. Basidiospores narrower (12.0–15.0 × 3.5–4.5 µm), known from North America…	*H. hortonii*
10′. Basidiospores wider (12.0–15.0 × 5–6 µm), known from Europe…	*H. depilatum*
